# Practical Notes and Formulæ

**Published:** 1880-12-20

**Authors:** 


### PRACTICAL NOTES AND FORMULÆ.

**Dyspepsia in Infants.**—Dr. Steiner recommends, n his Compendium of the diseases of children, the following formula, which he has often employed with good results in the treatment of dyspepsia in young children. Dyspepsia, the result of overloading the stomach with difficult digestible and badly assimilated foods, is a condition which is frequent in those who are brought up by hand. The treatment of such cases requires careful attention to diet, and where there is an excessive acidity of the stomach, magnesia and bicarbonate of soda may be employed as follows:

Soda bicarb, ................................0.20—0.50 centigrams.
Aq. destill................................ 80 grams.
Syrup simpl................................ 10 "

Sig.: A dessertspoonful every two hours.

In those cases in which there is excessive alkalinity on the other hand, acids in a very dilute form are specially indicated, and of these more especially hydrochloric acid:

Acic hydrochlor, dilute................................gtt. x.
Aq. destill................................70 grams.
Syrup simpl................................10 "

Sig.: A teaspoonful every two hours.

With very young children a dose of a centigram of pepsin may be administered before each meal.

The dyspepsia of older children, due to improper diet, can sometimes be quickly cured by an emetic and strict attention to diet. Colic of a dyspeptic character may often be cured by the employment of the following:

Soda bicarb................................0.50—0 80 centigrams.
Aq. fœnicul................................ 80 grams.
Syrup diacod................................ gtt. xv

Sig.: A teaspoonful every two hours.—*Therapeutic Gazette.*

**Pruritus Vulvæ.**—A writer in New York Medical Record says: I have found the applications of the balsam of Peru useful, of which I use the following:

℞ Pulv. gummi arabic................................ʒ ij.

Peruvian balsam................................ʒ j.

Oil of almonds................................ʒ jss.

Rosewater................................℥ j.

M. S. Apply freely with a camel's-hair brush, eight or ten times a day to the itching part.

This latter prescription, which was first suggested by Hufeland, I have also used for the past nineteen years, with the most happy reselts, for sore nipples, applied every hour for a few days. It has never failed in my hands to cure this troublesome affection. I prefer this prescription to the use of borax and alcohol, the nitrate of silver, the zinc ointment, or the saturated solution of borax, highly recommended by others.

**Desiccated Blood.**—The following formula for desiccated blood is used in the New York Hospital:

"Desiccated blood as thus prepared is completely and readily soluble in water at all temperatures below 160° F., and contains all the elements of blood except water and fibrin. The loss of the latter does not seem to impair its nutritive value, being but a very small proportion of the nitrogenous constituents of the blood."

A drachm of dried blood is necessary to represent a fluid ounce of blood of ordinary specific gravity.

℞ Sang. bov. exsicc.............................................ʒ vij.

Aqua..................................................℥ iv.

F. sol. et. adde..............................................

Glycerine..............................................

Spts. vini gallici, aa.............................................℥ j.

Sig.: A tablespoonful every three hours.

**Prescriptions in Disturbed Menstruation.**—In the Chicago Medical Gazette, Jan. 20, 1880, the following formula is recommended as a valuable remedy for a scanty, irregular menstruation, when associated with and dependent upon anæmia, neuralgia and neurasthenia:

℞ Tincturæ ferri chlor.............................................ʒ x.

Liquor potassæ arsenitas.............................................ʒ ij.

M. Sig.: Twelve drops after each meal, through a glass tube, in about one-third glass of water.

From the same source we are informed that the following prescription is specially serviceable in the various nervous manifestations which accompany the menopause:

℞ Sodii bromidi.............................................ʒ iv.

Tincturæ nucis vomicæ.............................................ʒ ij.

Elixir. calisayæ.............................................℥ ij.

Syrupi pruni virg.............................................ʒ j.

Elixir, simplicis.............................................ʒ vi.

M. Sig.: Two drachms, two, three or four times a day, as needed. —*Obstetric Gazette.*

**Remedy for Corns.**—Mr. Gezow, an apothecary of Russia, recommends the following, in the Pharmaceutische Zeitung, as a "sure" cure for corns, stating that it proves effective within a short time and without causing any pain. Salicylic acid, 30 parts; extract of cannabis indica, 6 parts; collodion, 240 parts. To be applied by means of a camel-hair pencil.—*Med. and Surg. Rep.*

**The Turkish Bath in Hay Asthma.**—A sufferer from Hay Asthma writes to the daily press of this city, that by taking a Turkish bath daily, from the time the first symptoms appeared, he has for three summers succeeded in almost wholly escaping this most annoying complaint. He, moreover, does not have a relapse when cold weather sets in, and so far from finding the baths debilitating, they prove decidedly tonic.—*Med. and Surg. Rep.*

**Formula for Sore Nipples.**—Dr. Howell recommends the following in the Canada Medical Record:

℞ Tannin..........................................ʒ j.

Sub-nit bismuth..................................ʒ ij.

Vaseline..........................................℥ j.

M. Sig.: To be applied constantly when the child is not nursing.

[The tincture of benzoin is an excellent application for sore nipples. It is harmtess to the child and painless to the mother. When applied it deposits a gummy coating which shields the excoriated surface, and which the child seldom removes in sucking.—ED. REC.] W.

**Preservative Fluid.**—The preservative fluid of the Grecian government is made up of: Alum, 100 parts; common salt, 25; nitre, 12; potass. carb., 60; arsenic, 10; and water 1,000 parts. Mix, cool and filter. Then add to ten parts of this fluid, by measure, four of glycerine and one of methylic alcohol. Bodies saturated in this will, it is claimed, keep for years their form, color and flexibility. The method is partly by injection and partly by immersion. The smaller objects of natural history collections, as snakes, birds, fruits, butterflies, algæ, etc., are preserved as perfectly as larger bodies. Dr. Wickerscheimer was the inventor of the process, the patent for which has been purchased by the government, and then by it the composition has been made public.—*Mich. Med. News.*

**Falling of the Hair.**—Mr. James Startin, in the British Medical Journal, suggests the following application in general loss of hair without obvious cause:

℞ Ung. petrolei..........................................

Ol. ricini, a a..........................................℥ ss

Hyd. ox. rub..........................................gr. v.

Liq. ammon. fort..........................................f ʒ ss.

Ol. rosmarini..........................................gtt. v.—M

**Only a Slight Gonorrhœa.**—Some time since a young lady of Richmond, Va., by some strange fatality stumbled upon the word *gonorrhœa.* She innocently asked the family physician its meaning. He told her that it was the technical name for headache j Being visited by a medical student regularly, who seemed very much in love with her, she, doubtless to show her aptitude at medical technicalities, (when at his next meeting he asked after her health), informed him that she had had a "slight gonorrhœa for the last four or five days!" He never came again, and she wonders why?—*Sou. Clinic.*

**Iodoform in Syphilictic Neuralgia.**—M. Mauriac speaks of a method usually employed with success by Prof. Zeiszl, of Vienna, to combat syphilitic neuralgia. He treats it with pills of iodoform, made according to the following formula:

℞ Pulv. iodoform..........................................grs. xx.

Ext. et pulv. gentiana..........................................q. s.

M. ft. pil. No. xx.
Sig.: Two or three pills daily.—*Jour. de Med. et de Chirurgie.*

**For Painful Hemorrhoids.**—

℞ Ext. belladonna........................................2 dr.

Iodoform........................................1 dr.

Acetate of lead........................................½ dr.

Vaseline........................................1 oz.

M.

S. Apply three or four times daily in small quantities.

The above will be found a most excellent application for painful or inflamed piles. The tumors should be bathed in cold water just before each application, and the bowels kept freely open with a gentle purgative.—*Med. Herald.*

**Treatment of Gaseous Dyspepsia**—In this form of dyspepsia, accompanied by fermentation with the rapid disengagement of large volumes of gas after meals, the most satisfactory remedy is chloroform, in the dose of fifteen to twenty drops in a little syrup. After a few moments the gas is expelled from the stomach and fermentation arrested.

**Chinese Varnish.**—This is prepared by mixing three parts of freshly-beaten defibrinated blood with four parts of slacked lime and a little alum. The thin-liquid mass may be used at once. Pasteboard coated with it is said to become as hard as wood. Straw baskets may be rendered by it water and oil tight.—*Polyt. Notizbl.*

**Hemorrhoids Treated with Capsicum.**—In cases of hemorrhoidal congestion, Vidal regards capsicum annum as the best remedy as the best remedy. He prescribes four or five pills daily, each containing 20 centigrams, half at breakfast time and half at supper time. Under this influence the congestion and all the painful symptoms which accompany it disappear rapidly.—*Jour. de Med.*

**Treatment of Diphtheria.**—M. Crequy, according to La France Medicale, commences his treatment of diphtheria by removing the false membrahe with a forceps; he endeavors by a twisting motion to remove the membrane, without breaking it, in as large a piece as possible; he then with a sponge dabs the denuded mucous surface with a solution of tannin. He never hesitates to adopt this method in all cases.—*Med. and Surg. Rep.*

**For the Cure of Tubercular Laryngitis.**—Dr. Wm. Pepper gives the following prescription:

℞ Tr. benzoici comp........................................fl. ʒ ij.

Glycerine........................................fl. ℥ ss.

Aquæ........................................fl. ℥ iv.

Sig.: To be used as a gargle.—*Canada Med. Rec.*

**Disguising the Taste of Epsom Salts.**—According to the Gaz. des Hop, June 12, 1880, the purgatif Yvon consists of sulphate of magnesia twenty grams, water forty grams, and essence of mint two or three drops. The essence of mint completely masks the disagreeable taste of the sulphate, providing that the quantity of the vehicle is inconsiderable.—*Med. and Surg. Rep.*

